# Mutational profiling in acute lymphoblastic leukemia by RNA sequencing and chromosomal genomic array testing

**DOI:** 10.1002/cam4.4101

**Published:** 2021-07-20

**Authors:** Cecilia Yeung, Xiaoyu Qu, Olga Sala‐Torra, David Woolston, Jerry Radich, Min Fang

**Affiliations:** ^1^ Fred Hutchinson Cancer Research Center Clinical Research Division Seattle WA USA; ^2^ University of Washington Seattle WA USA; ^3^ Seattle Cancer Care Alliance Seattle WA USA

**Keywords:** leukemia, molecular pathology, next‐generation sequencing, RT‐qPCR

## Abstract

**Background:**

Comprehensive molecular and cytogenetic profiling of acute lymphoblastic leukemia (ALL) is important and critical to the current standard of care for patients with B‐acute lymphoblastic leukemia (B‐ALL). Here we propose a rapid process for detecting gene fusions whereby FusionPlex RNA next‐generation sequencing (NGS) and DNA chromosome genomic array testing (CGAT) are combined for a more efficient approach in the management of patients with B‐ALL.

**Methods:**

We performed RNA NGS and CGAT on 28 B‐ALL samples and, in four patients, compared fixed cell pellets to paired cryo‐preserved samples as a starting material to further assess the utility of cytogenetic fixed pellets for gene expression analysis.

**Results:**

Among the fixed specimens, when using alternative techniques as references, including karyotype, fluorescence in situ hybridization, CGAT, and RT‐qPCR, fusions were detected by RNA NGS with 100% sensitivity and specificity. In the four paired fixed versus fresh cryopreserved samples, fusions were also 100% concordant. Four of the 28 patients showed mutations that were detected by RNA sequencing and three of four of these mutations had well‐known drug resistance implications.

**Conclusions:**

We conclude that FusionPlex is a robust and reliable anchored multiplex RNA sequencing platform for use in the detection of fusions in both fresh cryopreserved and cytogenetic fixed pellets. Gene expression data could only be obtained from fresh samples and although limited variant data are available, critical hotspot variants can be determined in conjunction with the fusions.

## Graphical abstract

This study describes a novel RNA next‐generation sequencing assay used in tandem with chromosomal genomic array testing upon fixed cell pellets typically used for cytogenetic studies to detect the molecular profiles of B‐acute lymphoblastic leukemia.

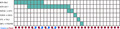

## 1 INTRODUCTION

Acute lymphoblastic leukemia (ALL) is the most common cancer in children, with a majority being of B‐cell origin, some of T‐cell origin, and a rare subset which is of mixed lineage. Although B‐ALL is more common in children (~80%), pediatric patients with B‐ALL have a very high remission rate of around 98%. B‐ALL in adults, however, carries a much worse prognosis and most deaths from ALL are seen in adult patients. Comprehensive molecular and cytogenetic profiling of ALL is critical to the current standard of care for both adult and pediatric ALL, as these genetic aberrations drive prognostication, risk‐stratification, treatment options and can identify markers used for tracking the minimal residual disease.

Classification of ALL starting with the 2008 World Health Organization (WHO) was based on recurrent genetic abnormalities,^1^ and was further refined and expanded in the latest version of the 2016 WHO hematologic malignancy definitions.^2^ The variety of relevant genetic lesions in B‐ALL is extensive. For example, genes in the B‐cell development pathway are altered in nearly 90% of Philadelphia chromosome‐like (Ph‐like) ALL patients. Small deletions are common in B‐ALL, often seen with *CRLF2* rearrangements and inactivating mutations of the JAK/STAT (*IL7R*, *FLT3*, *SH2B3*, *JAK1*, and *JAK3*) or RAS signaling pathways (*NF1*, *KRAS*, *PTPN11*, and *BRAF*). Recurrent gene fusions are seen in 93% of CRLF2‐overexpressed Ph‐like ALL patients,^3^ with many fusions inducing small deletions at the breakpoint that are detectable by high‐resolution chromosome genomic array testing (CGAT), also known as single nucleotide polymorphism (SNP)‐array comparative genomic hybridization. *IKZF1* alterations (including both point mutations and deleterious deletions) is the most common mutation seen in Ph‐like ALL, with other cases showing mutations and/or copy number alterations (CNA) in *ETV6*, *EBF1*, *PAX5*, *TCF3*, *ERG*, *RAG1*/*2*, *BLNK*, *BCL11A*, *IKZF2*, *LEF1*, *MEF2C*, *SOX4*, and *SPI1* ^4^ that are detectable by next‐generation sequencing (NGS). Moreover, hypodiploid B‐ALL has two distinct subtypes of high‐risk B‐ALL frequently misdiagnosed by conventional cytogenetics and fluorescence in situ hybridization (FISH). Low‐hypodiploid ALL typically harbors alterations of *TP53*, *RB1*, and *IKZF2*. Near haploid ALL displays a high frequency of *IKZF3* mutations and alterations targeting the RTK and RAS signaling pathways, as well as deletions of a histone cluster at chromosome 6p22.^5^, ^6^ In these high‐risk subtypes, CGAT may be used as a surrogate to identify Ph‐like signatures.

As the relevant genetic diagnostics needed for the risk assessment of ALL have broadened to include cytogenetics, gene fusions, mutations, and gene expression, the challenge to rapidly perform these diagnostics has become apparent. A comprehensive testing algorithm is difficult to develop and commonly requires multiple high complexity testing strategies with long turnaround times. Here we propose a rapid process by which anchored multiplex fusion detection by RNA NGS and CGAT can be combined for a more efficient approach in the management of patients with B‐ALL.

## 2 MATERIALS AND METHODS

### 2.1 Patients and tissue samples

**FIGURE 1:**
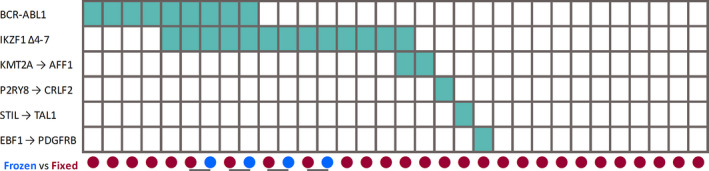
Summary view of samples and fusions. All fusions detected in this study are displayed on the left. In the grid, a green rectangle denotes the fusion was detected by next‐generation sequencing in a given sample. Below the grid, the red disks denote methanol/acetic acid fixed cells, while the blue disks denote cryopreserved specimens. A total of 32 specimens were tested, representing 28 acute lymphoblastic leukemia patients, among whom four patients were tested using both fixed and cryopreserved specimens. Horizontal lines denote that the two samples were from the same patient

Acute lymphoblastic leukemia samples for which CGAT was performed between 2011 and 2017 in our institution were identified for the current study. Those with at least two vials of fixed cells as a leftover from clinical testing were used. RNA was extracted using ReliaPrepTM RNA Cell Miniprep System (Promega) from 32 specimens representing 28 ALL samples (Figure 1). One fixed cell pellet each for 24 patients was used, and paired cryo‐preserved and fixed specimens were used for four additional patients. Fixed specimens were leftover pellets from cytogenetic workup with standard methanol/acetic acid fixation that had been stored at −20°C for 1–5 years. Blast percentages (blast%) ranged from 2.6% to >80%.

RNA concentration was measured using Qubit RNA HS Assay Kit (Thermo Fisher). Hundred nanograms of RNA were used for each of the 32 RNA samples from 28 patients. RNA quality was assessed with PreSeq RNA QC Assay following procedures recommended by ArcherDX (CO). Briefly, complementary DNA (cDNA) was diluted 1:10 and amplified with iTaq SYBR Green Supermix (Bio‐Rad Laboratories) and VCP primer mix (ArcherDX). The reaction assesses the amount of RNA fragments greater than 100bp and the cycle threshold (Ct) values are intended to be tracked over time for each lab to establish rejection criteria regarding RNA quality.

Complementary DNA quality was assessed with a NanoDrop 2000 Spectrophotometer (Thermo Scientific) and visualized on a 1% agarose gel with ethidium bromide to detect/exclude degradation. The criteria for acceptable cDNA quality included visible bands by 1% agarose gel and spectrophotometer reading by A260 nm/A280 nm ratio of 1.4 to 2.

### 2.2 Karyotype and FISH analysis

Marrow aspirate or peripheral blood samples from patients were tested for cytogenetic abnormalities using clinically validated protocols at Seattle Cancer Care Alliance (SCCA) for standard culturing, G‐banding karyotyping, and FISH. Karyotype designation was based on the International System for Human Cytogenetic Nomenclature (ISCN 2016).^7^ FISH probes were purchased from Abbott Molecular (Abbott Park, IL) and Cytocell‐Rainbow Scientific. FISH probes used in this study targeted 4p, 5p, 7cen, 7q, 8cen, 8q, 9p, 12p, 13qter, 14q, 17p, 21q, 22q, *BCR*‐*ABL1*, *KMT2A* (*MLL*), and *IgH*. Genes where FISH probes were not available to include *IKZF1*, *STIL*, *TAL1*, *P2RY8*, and *CRLF2*.

### 2.3 Reverse‐transcription PCR and Sanger sequencing

The reverse‐transcription polymerase chain reaction (RT‐PCR) and Sanger sequencing validation for the *P2RY8*‐*CRLF2* fusion RNA assay were reverse transcribed using SuperScript IV Reverse Transcriptase (Thermo Fisher) and PCR amplified using primers targeting *P2RY8* (NM_178129.4 Exon 1: 5′‐TTAAGCGTTGCATCCTGTTA‐3′) and *CRLF2* (NM_022148.3 Exon 2: 5′‐TCAGGTTGGTCCTGGAGTAT‐3′). The PCR product was then Sanger sequenced at the Genomics core of Fred Hutchinson Cancer Research Center.

### 2.4 Fusion testing with anchored multiplex PCR NGS

Libraries were prepared using the FusionPlex ALL kit (ArcherDX) targeting 44 fusion genes with target regions detailed in Supporting Information S1 and sequenced on a MiSeq (Illumina). Quality control (QC) of the library was acceptable if PreSeq Ct values were less than 30. Library concentrations were quantified using the KAPA Library Quantification Kits (Roche). An average of >850,000 base pairs of nucleotides was sequenced at a depth of >500× coverage. Fusion, oncogenic isoform, single nucleotide variant (SNV), and insertion/deletion (Indel) variant data were analyzed using Archer Analysis 6.0 software, and RNA expression data were analyzed using Archer Analysis 5.1 software. Only fusions and oncogenic isoforms identified as “Strong” by Archer Analysis' default user settings (defined as at least five breakpoint spanning reads, presence in the Quiver database, and at least 10% GSP2 reads spanning the breakpoint) were considered in this study. Within the Archer Analysis 5.1 software, SNV and Indel Variants were initially filtered by alternate observation (AO) value (≥10), unique AO (UAO) value (≥5), expressed allele fraction (AF) value (≥0.05), global population AF from the genome aggregation database (gnomAD) value (<0.05), and quality score (>100). We chose these parameters with consideration of the minimum recommendations from the manufacturer and slightly increased the stringency to a level comparable to other similar NGS assays in our laboratory. Only filtered SNV/Indel alterations that were listed within the Archer FusionPlex assay targets panel and which had the following consequences and pathogenicity based on the NCBI ClinVar or COSMIC databases were retained for this study: allele‐specific point mutations, frameshifts, missense, loss of start or stop codon, splice site variants, variants leading to transcript amplification or ablation, and likely pathogenic or pathogenic consequence. Variant calls with positive or indeterminate Sequencing Direction Bias were omitted. Although filtered by gnomAD, SNV calls were further verified with NCBI's dbSNP collection, 1000 Genomes Project Phase 3 Browser, and/or the Ensembl GRCh38 database. The final set of filtered variants was visually inspected with Integrative Genomics Viewer. RNA expression profiling was performed per Archer Analysis software without further modification.

### 2.5 Chromosome genomic array testing

Chromosome genomic array testing was performed for the detection of DNA CNA or copy‐neutral loss of heterozygosity (cnLOH) by SNP genotyping along with non‐SNP copy number probes using CytoScan HD (Affymetrix) according to the manufacturer's protocol. In general, CGAT was performed only on specimens with 20% or more tumor cells. Specimens with low tumor content (<20%; Patients 15, 21, and 27) were flow‐sorted to enrich the abnormal lymphoblast population to greater than 50% prior to CGAT. The size filter for an abnormal call was 100 kb for CNA and 10 Mb (and terminal) for cnLOH.

## 3 RESULTS

### 3.1 Patient demographics

The characterizations of patients and samples are shown in Table 1.

**TABLE 1:** Quality metrics and demographics of patients and their samples

Patient characteristic	Number of patients
Diagnoses	
T‐ALL	3
B‐ALL (Ph+)	7
B‐ALL (Ph−)	18
Cytogenetic subgroups	
Normal karyotype	8
Abnormal karyotype	19
N/A	1
Demographics	
Age (median)	20–77y (median 49y)
Gender (M:F)	18:10
Clinical course	
New diagnosis	2
Post‐treatment	2
Relapsed refractory	6
Post‐salvage, pre‐CAR‐T	4
Unknown	14

Abbreviations: B‐ALL, B‐cell acute lymphoblastic leukemia; CAR‐T, chimeric antigen receptor T cell; Ph, Philadelphia chromosome (a.k.a. *BCR*‐*ABL1*); T‐ALL, T cell acute lymphoblastic leukemia.

### 3.2 RNA‐NGS and existing clinical assays detected anomalies in samples with a wide range of tumor content

**FIGURE 2:**
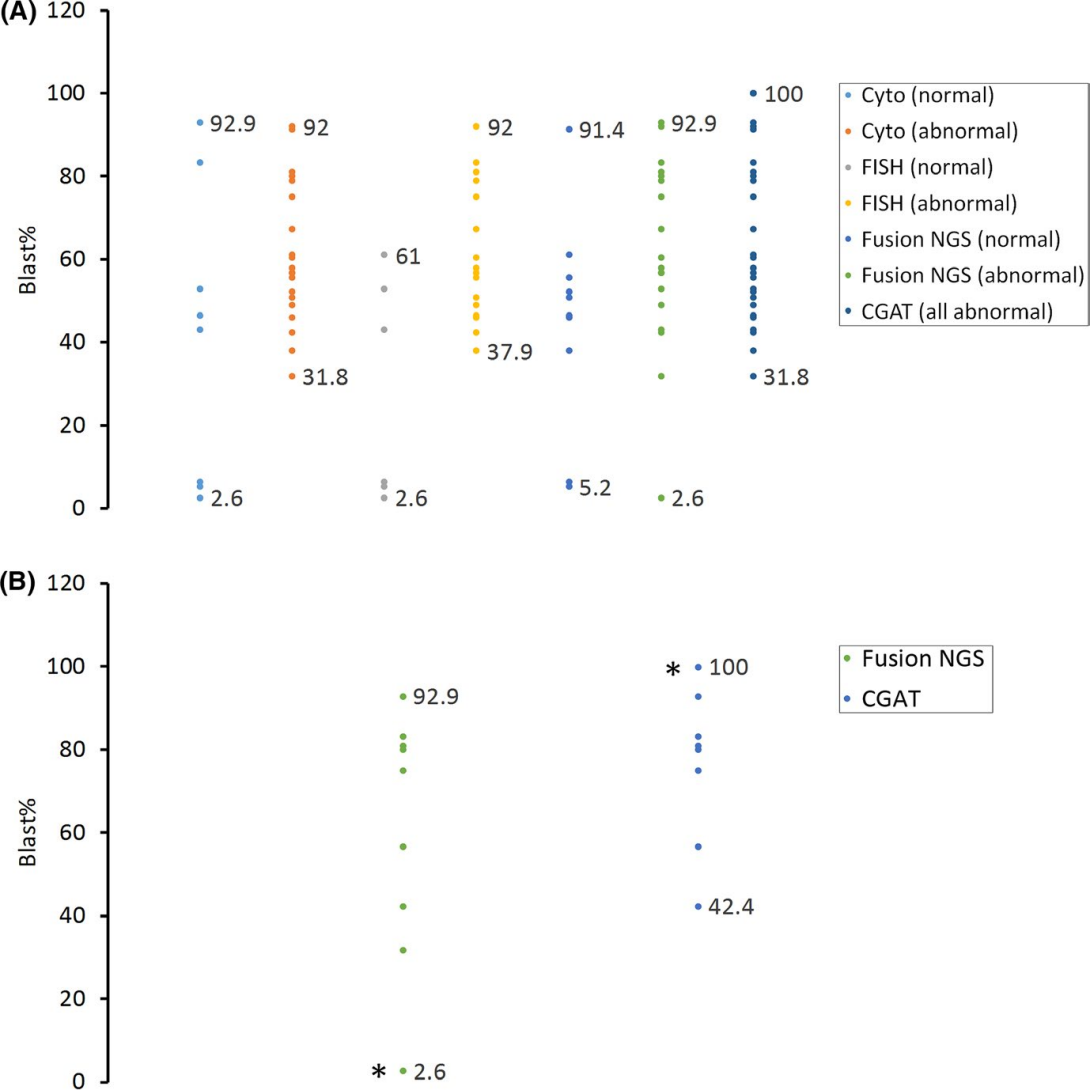
The range of tumor burdens at which various assays reported abnormal results. The tumor burden was estimated using the abnormal blast% reported by the concurrent flow cytometry study. The data labels display the lowest and the highest blast % within the sub‐group. Karyotype, fluorescence in situ hybridization (FISH), and fusion next‐generation sequencing (NGS) were performed on fixed cell pellets. Chromosomal genomic array testing (CGAT) was performed on DNA extracted from the fresh specimen. (a) The comparison for all four tests using all results; (b) the comparison between fusion NGS and CGAT on the detection of *IKZF1* Δ4‐7. Asterisks denote the same bone marrow specimen for which CGAT was performed on flow‐sorted abnormal blasts, while fusion NGS was performed on whole marrow

Comparison of the different diagnostic platforms for the range of tumor burdens where abnormal results could be detected was reviewed for the limit of detection (Figure 2). The tumor burden was estimated using the abnormal blast% reported by each patient sample's concurrent clinical flow cytometry study. Karyotype, FISH, and fusion NGS were performed on fixed cell pellets and could detect malignancy in samples with tumor burden as low as 2.6%. In this study, consistent with our general clinical practice, CGAT was performed only on specimens with 20% or more tumor cells and on flow‐sorted abnormal lymphoblast cells when necessary. The CGAT sample with the lowest tumor content, Patient 4, had 31.8% abnormal blasts.

### 3.3 RNA‐NGS detected fusion transcripts associated with ALL

**FIGURE 3:**
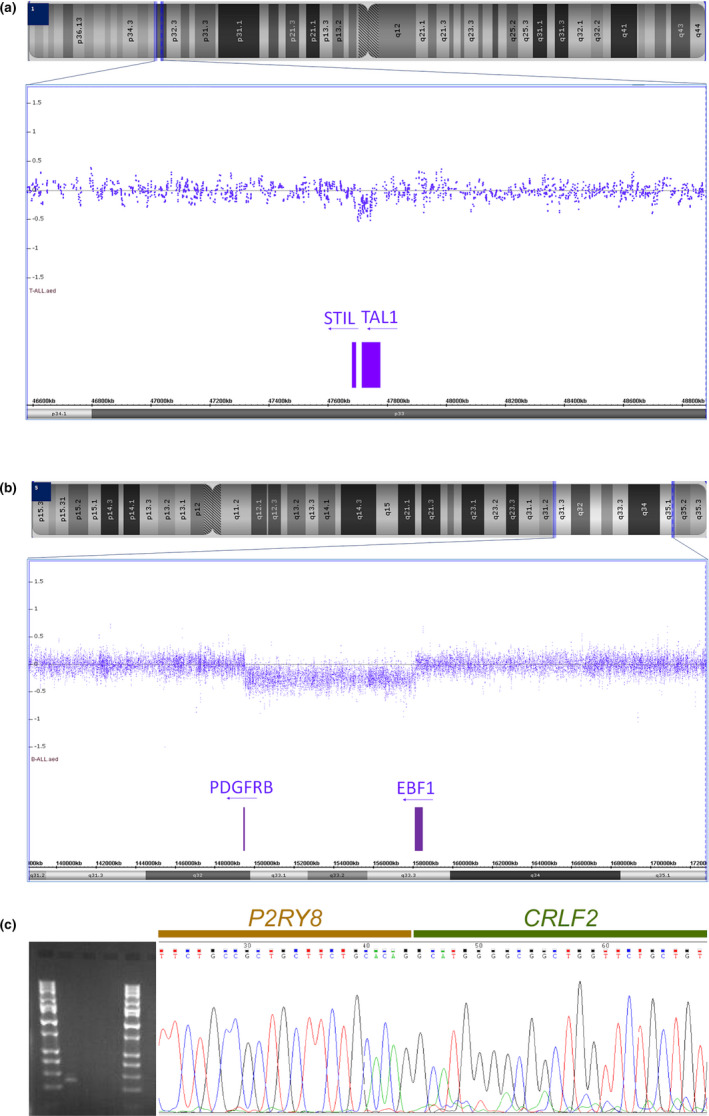
Next‐generation sequencing detected fusions that were suspected using chromosomal genomic array testing (CGAT) data: (A) A 40‐kb deletion at approximately 40% was minimally visible by CGAT between *STIL‐TAL1*; and (B) an 8.4 Mb deletion with breakpoints located at the *PDGFRB* and *EBF1* loci. The graphs display CytoScan HD array results visualized using Chromosome Analysis Suite (ChAS; Thermo Fisher Scientific). (C) *P2RY8‐CRLF2* fusion in Patient 17 was verified by PCR (gel on left) and Sanger sequencing (right)

**FIGURE 4:**
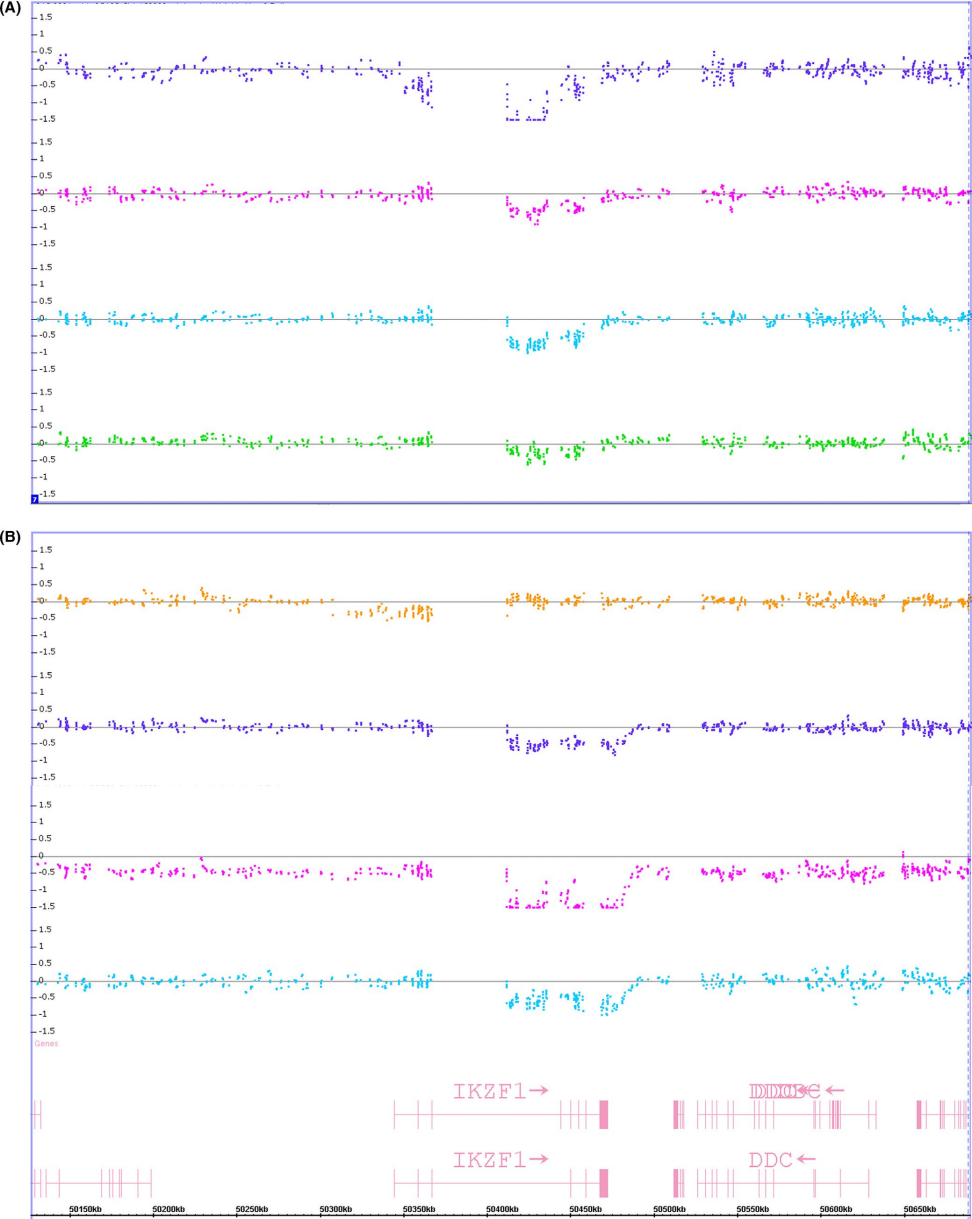
Log2 ratio of copy number probes in samples with different types of *IKZF1* deletions. (A) Samples with *IKZF1* Δ4‐7 detected by fusion next‐generation sequencing (NGS) and deletion at the gene locus detected by chromosomal genomic array testing (CGAT); and (B) samples with *IKZF1* deletion detected by CGAT but not *IKZF1* Δ4‐7 by fusion NGS. The graphs display weighted log2 ratio of copy number probes of CytoScan HD array visualized using Chromosome Analysis Suite (ChAS). The locations of the gene and exons are displayed at the bottom of the panel (B)

Among the 28 samples, 17 were shown to have ALL‐related fusions and oncogenic isoforms, including *BCR*‐*ABL1* (*n* = 7), *IKZF1* Δ4‐7 (*n* = 9), *KMT2A*‐*AFF1* [*t*(4; 11), *n* = 2], *EBF1*‐*PDGFRB*, *P2RY8*‐*CRLF2*, and *STIL*‐*TAL1* (*n* = 1 each for the last three). Among all samples, CGAT results were available in all, karyotype data in 26 samples, and FISH data in 22 samples. One patient had neither karyotype nor FISH data available for review. Based on the comparison between fusion calls by NGS and the existing clinical techniques in our lab, we present the following data groups:

1. Fusions were detected by NGS and one other technique. These included *BCR*‐*ABL1* (*n* = 7), *KMT2A*‐*AFF1* [*t*(4; 11), *n* = 2], and *EBF1*‐*PDGFRB* (*n* = 1). (Table 2: Comparative karyotype/chromosomal genomic array testing [CGAT]/fusion next‐generation sequencing [NGS] data). The *BCR*‐*ABL1* and *KMT2A*‐*AFF1* rearrangements both involve microscopically visible chromosomal translocations, which were also verified by FISH. The *EBF1*‐*PDGFRB* fusion in Patient 28 was first inferred from an 8.4 Mb deletion of chromosome 5q23q33 detected by CGAT (Figure 3b). The deletion was not visible by karyotype but was confirmed by FISH.
2. Fusions uniquely identified by NGS, including *STIL*‐*TAL1* and *P2RY8*‐*CRLF2* (*n* = 1 each). The *STIL*‐*TAL1* fusion in Patient 13 was minimally visible by CGAT (Figure 3a) and was not targeted by the conventional FISH panel. Patient 17 carries the *P2RY8‐CRLF2* fusion, a cryptic rearrangement not visible by karyotype and not tested by FISH. The submicroscopic deletion that leads to *P2RY8‐CRLF2* fusion is theoretically over 300 kb in size. However, there are significant gaps in probe coverage within this region by the CGAT assay and the existing probes can have noisy data. The fusion was not clearly discernable by CGAT in this sample. We validated the *P2RY8*‐*CRLF2* fusion by follow‐up RT‐PCR with the fusion junction sequence confirmed by Sanger sequencing (Figure 3c).
3. *IKZF1* oncogenic isoform, sometimes discordant between NGS and CGAT (Table 2). Among 28 samples, nine samples showed *IKZF1* oncogenic isoforms by RNA‐NGS, including seven with a focal genomic deletion called out by CGAT and two without. Meanwhile, four samples were demonstrated to have the focal genomic deletion at the *IKZF1* locus on CGAT, but *IKZF1* oncogenic isoform was not detected by NGS. Close examination of the array results revealed that the seven samples with concordant NGS and CGAT results for *IKZF1* had deletions that did not impact the first or the last exon of the gene (see examples in Figure 4a). Therefore, the introgenic deletion likely leads to exon‐skipping. The blast% in these seven samples ranged from 56.7% to 100% per concurrent clinical flow cytometry reports. The specimen with a blast% of 100% refers to the CGAT specimen for Patient 15, which was flow‐sorted by abnormal blast% (fusion NGS was performed on the left‐over fixed cell pellet from the whole bone marrow). The high abnormal blast% in these specimens likely enabled the detection of the deletion by CGAT despite its small size (<100 kb). The two samples with *IKZF1* oncogenic isoform by NGS and no definitive abnormality at the *IKZF1* locus had lower blast%: Patient 4 had 31.8%, while Patient 12 had 42.4% abnormal blasts by flow cytometry. For the four samples with *IKZF1* deletion by CGAT but no *IKZF1* oncogenic isoform called by NGS, the deletions disrupted either the first or last exon of the gene (Figure 4b). These are unlikely to yield exon‐skipping events but result in haploinsufficiency of the gene.

**TABLE 2:** Comparative karyotype/chromosomal genomic array testing (CGAT)/fusion next‐generation sequencing data

Patient code	Clinical indication	Sample blast %	Karyotype	Abnormal FISH	Potential Ph‐like deletions^a^ by CGAT	Fusions called by Archer	*IKZF1* deletions called by Archer
1	T‐ALL	61	49,XX,add(10)(p11.2),+3mar[14]/46,XX[6]	None detected	None detected	None detected	None detected
2	B‐ALL	67.3	46,XX,−9, t(9;22) (q34;q11.2), +der(22) t(9;22)[1]/48,idem,+der(22)t(9;22)x2[13]/ 46,XX[6]	*BCR*/*ABL1* rearrangement (64.5%)	*IKZF1*, *PAX5*(LS), *VPREB1*(LS)	*BCR*→*ABL1*	None detected
3	B‐ALL	37.9	56,XX,+X,+X,t(3;?)(p10;?),+6,+10,+15,−17, der(17)t(17;17)(p13;q12),+18,+19,+21, +2mar[10]/56~57,idem,‐t(3;?),+8,‐der(17)[cp2] /46,XX[8]	+8q (41.5%), homozygous 9p‐ (44.5%), and −17 (43.0%)	None detected	None detected	None detected
4	B‐ALL	31.8	46,XX,add(2)(p12),add(2)(q11.2),del(6)(q15),+10,add(12)(p11.2),add(14)(q?24),der(14) t(2;14)(q?13;q24),−15,add(17)(q23),del(18) (q21.1),−22,+der(?)t(?;2)(?;p11.2)[7]/ 46,XX[13]	Not performed	*EBF1*	None detected	Yes
5	B‐ALL, post‐chemo for relapse	55.7	45~46,XX,t(1;14)(p22;q24),−4,i(7)(q10), del(8)(p12),der(12)t(4;12)(q13;p12),add(14) (q32),del(17)(p11.2),add(20)(q13.1)[cp3]/ 45,idem,‐t(1;14),‐i(7),+add(7)(p10)[2]/ 46,XX,t(1;1)(p36.1;q21),der(7)i(7)(q10)t(7;11) (q22;q13),del(8),?add(12)(p12),add(14), add(20)[1]/46,XX[20]	+7q (38.5%), 9p‐ (43.5%), and 17p‐ (23.3%)	*IKZF1*(LS)	None detected	None detected
6	B‐ALL, refractory	52.2	47,X,r(?Y),+1,add(1)(p13),del(1)(q21),add(7)(q34),−10,del(11)(q13q23),add(14)(p11.2), +mar[cp3]/ 46,XX[17]	Not performed	*IKZF1* (70 kb)	None detected	None detected
7	B‐ALL, relapse	92.9	46,XY[20]	Not performed	*EBF1*, *IKZF1*	None detected	Yes
8	B‐ALL relapse	91.4	45,XY,−7,t(9;21)(p22;q22)[cp11]/45,sl,t(3;11) (q21;p15)[2]/46,XY[7]	Not performed	None detected	None detected	None detected
9	B‐ALL, relapse	46	37,XX,−2,−3,−4,−7,−9,−12,−15,−16,−17[6]/37,sl, add(6)(p21.3)[2]/67~74,slx2,−5,−8,−10,−13,−14, −22[cp6]/46,XX[6]	3q‐ (33.0%), +8q (20.7%), 9q‐ (28.0%), and +22q (8.3%);	*IKZF1*(LS)	None detected	None detected
10	B‐ALL	49	46,XY,t(9;22)(q34;q11.2)[2]/47,sl,+5,+der(22) t(9;22)[1]/62,sdl1,+X,+Y,+2,+4,+8,+10,+11, +14,+15,+16, +17,+21,+21,+21[10]/46,XY[7]	+4p/+14q (19%), −7 (5.8%), +8 (10%), and +14q or *IGH* rearrangement (11%)	*IKZF1*(LS) <10%	*BCR* →*ABL1*	None detected
11	B‐ALL	81	46,XY,t(9;22)(q34;q11.2),del(12)(p12)[10]/ 46,XX[10]	Not performed	*IKZF1*, *PAX5*(102 kb), *VPREB1*	*BCR* →*ABL1*	Yes
12	B‐ALL diagnosis	42.4	46,XY,del(2)(p21p23)[5],del(3)(p21p23)[5], r(9)(p12p24)[4],t(9;22)(q34;q11.2)[9],i(17) (q10)[4][cp9]/46,XY[11]	*BCR*/*ABL1* rearrangement (27.3%) and 9q‐ (21.0%)	VPREB1	*BCR* →*ABL1*	Yes
13	T‐ALL	52.9	46,XY[20]	None detected	50kb deletion between *STIL* and *TAL1*	*STIL* →*TAL1*	None detected
14	B‐ALL	83.3	Not performed	9p‐ (76.5%)	*IKZF1*, *VPREB1*	None detected	Yes
15	B‐ALL, pre‐CAR‐T	2.6	46,XY[20]	None detected	*IKZF1*	None detected	Yes
16	B‐ALL, pre‐CAR‐T	80	46,XY,t(2;19)(p21;?p13.1)[1]/46,XY[20]	Not performed	*IKZF1*, *PAX5* (2 Mb subclone)	None detected	Yes
17	B‐ALL	43	46,XY[20]	None detected	None detected	*P2RY8* → *CRLF2*	None detected
18	B‐ALL	56.7	Not performed	+8q (3.8%), *BCR/ABL1* rearrangement (61.5%), and +17q (44.5%)	*IKZF1*	*BCR* →*ABL1*	Yes
19	B‐ALL, relapsed	58	46,XY,t(9;22)(q34;q11.2)[4]/46,XY[16]	*BCR*/*ABL1* rearrangement (2.4%); +21q (9.6%)	None detected	*BCR* →*ABL1*	None detected
20	B‐ALL, pre‐CAR‐T	79	45,XY,del(7)(p11.2),add(8)(p12),−10,t(11;13) (p12;q14)[cp12]/46,XY,t(1;16)(q12;p13.1)[4]/46,XY[4]	12p‐ (75.5%) and 17p‐ (17.7%)	*EBF1*, *IKZF1*, *PAX5*(3.4 Mb), *TCF3* (502 kb, subclone)	*ETV6* → LOC101928224 transcriptional read‐through	None detected
21	B‐ALL, pre‐CAR‐T	6.4	46,XY[20]	None detected	*EBF1*, *IKZF1* (74 kb), *PAX5*(267 kb)	None detected	None detected
22	B‐ALL, post‐consolidation	75	48,XY,t(4;11)(q21;q23),+6,+7,i(7)(q10)[16]/ 46,XY[4]	*KMT2A* (*MLL*) rearrangement (58.5%)	*IKZF1*	*KMT2A* → *AFF1*	Yes
23	B‐ALL	46.4	46,XY[20]	+1q (16.0%) and IGH rearrangement with partial 5'IGH deletion of the other chr.14 homologue (35.0%);	*EBF1*, *PAX5*(246 kb)	None detected	None detected
24	B‐ALL	92	46,XX,t(4;11)(q21;q23),t(11;13)(q23;q12)[10]/46,XY[10]	*KMT2A* (*MLL*) rearrangement [11q23] (81.5%)	None detected	*KMT2A* → *AFF1*	None detected
25	T‐ALL	50.8	49,XY,del(9)(q13),der(14)t(5;14)(p12;p13), +marx3[14]/46,XY[6].ish mar(163C9++)	+5p (60.5%) and 13qter amplification (67.0%)	None detected	None detected	None detected
26	B‐ALL, relapse	56.8	46,XY,t(9;22)(q34;q11.2)[12]/46,sl,del(7) (p11.2)[4]/46,XY[4]	Not performed	None detected	*BCR* →ABL1	None detected
27	B‐ALL	5.2	46,XX[20]	None detected	None detected	None detected	None detected
28	B‐ALL, new diagnosis	60.4	46,XX,add(9)(p24)[2]/46,XX[18]	deletion of 5’ *PDGFRB* (54.5%) suggesting *PDGFRB* rearrangement;	None detected	*EBF1* → *PDGFRB*	None detected

Abbreviations: B‐ALL, B‐cell acute lymphoblastic leukemia; CGAT, chromosomal genomic array testing; FISH, fluorescence in situ hybridization; LS, long stretch / part of a long stretch of deletion; Ph‐, Philadelphia chromosome (a.k.a. *BCR*‐*ABL1*); T‐ALL, T cell acute lymphoblastic leukemia.

^a^ Deletions were either focal and encompassed a single gene or a few exons, or part of a long stretch of deletion (LS) encompassing multi‐Mb regions beyond the denoted gene.

Taken together, among all samples where an alternative testing method revealed gene fusions (*n* = 12), including *BCR*‐*ABL1* (*n* = 7 by karyotype and/or FISH), *KMT2A* (*MLL*, *n* = 2 by FISH and karyotype), *EBF1*‐*PDGFRB* (*n* = 1 by CGAT and FISH), *P2RY8*‐*CRLF2* (*n* = 1 by RT‐qPCR), and *STIL*‐*TAL1* (*n* = 1 by CGAT), corresponding fusion transcripts were successfully identified by RNA NGS. Among all the samples where no gene fusion was indicated, none had fusion transcripts reportable by RNA NGS. Hence, RNA NGS detected gene fusions with 100% sensitivity and specificity. These calculations exclude data from the *IKZF1* oncogenic isoform because CGAT does not distinguish between deletions that lead to oncogenic isoform versus haploinsufficiency and is, therefore, not an appropriate reference for RNA NGS assay performance evaluation in this regard.

### 3.4 Comparison of assay performance between paired frozen and fixed samples

**FIGURE 5:**
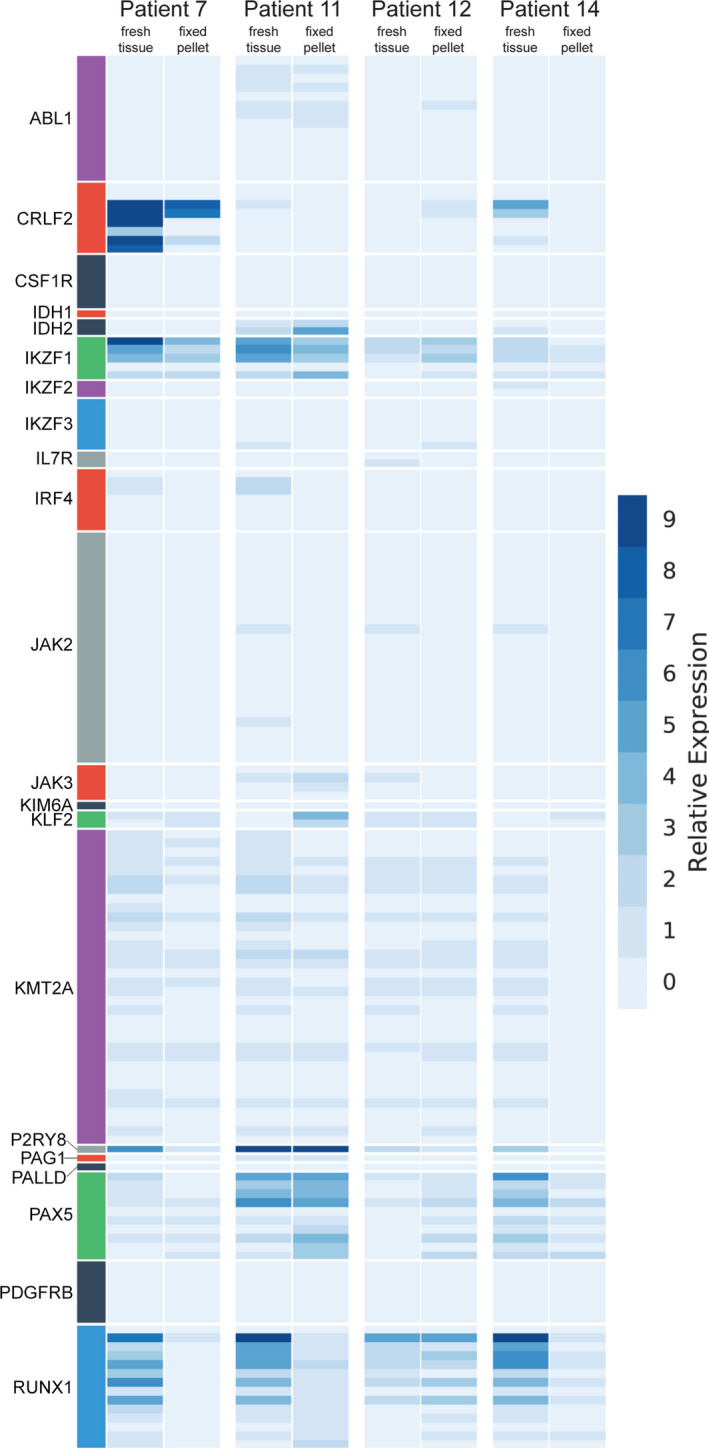
RNA expression of 21 selected genes in the paired cryopreserved versus fixed pellet samples (fixed using methanol/ascetic acid fixation). The heat map suggests a low correlation between the paired samples—that using fresh tissue to assess RNA expression facilitates a more sensitive test by avoiding the nucleic acid degradation caused by fixation

The comparison of RNA expression assay performance between the four paired fresh‐frozen and fixed pellet samples showed that the fixed specimens are appropriate for fusion and oncogenic isoform detection by NGS, but not for expression profiling. The PreSeq RNA QC Assay Ct values and library yield for the paired frozen and fixed samples were similar (Supporting Information S2). The two sample matrixes showed consistent fusion and oncogenic isoform calls in all four samples (Figure 1). However, the expression results were not consistent (Figure 5).

### 3.5 Mutation and expression data supported Ph‐like signatures

The expression of Ph‐like genes (such as *CRLF2*, *IKZF1*, *RUNX1*) was increased in three of the four fresh cases used within the RNA expression assay. Also, hotspot mutations were identified in 26/28 cases with the earlier‐described filtration criteria. However, the FusionPlex Heme v2 RNA sequencing assay was described by the manufacturer to reliably detect mutations in RNA at the hotspots outlined in Supporting Information S3. Therefore, we further filtered the variants called and only retained the mutations denoted by the manufacturer's product insert. After the application of all filtration parameters, only four patients showed variants that could be reliably called by RNA sequencing. These are detailed in Table 3. Mutations seen were notably in *JAK2*, *ABL1*, and *NOTCH1*.

**TABLE 3:** Variants detected in our patients after the above filtration criteria were applied. Mutations seen were notably in *JAK2*, *ABL1*, and *NOTCH1*

Patient	Gene	HGVSc	HGVSp
13	*NOTCH1*	NM_017617.3:c.4754T>C	p.(Leu1585Pro)
20	*JAK2*	NM_001322194.1:c.2047A>G	p.(Arg683Gly)
23	*JAK2*	NM_001322194.1:c.2047A>G	p.(Arg683Gly)
26	*ABL1*	NM_005157.4:c.949T>C	p.(Phe317Leu)

Abbreviations: HGVSc, human genome variation society; HGVSp, human genome variation society protein.

## 4 DISCUSSION

This study demonstrates that FusionPlex is highly sensitive and specific in detecting recurrent fusions in ALL from fixed archived cytogenetic cell pellets as well as cryopreserved samples from clinical patients with well‐annotated disease profiles and tumor burdens. Having achieved 100% sensitivity and specificity, the data suggest that FusionPlex NGS is complementary to current clinical methods of genetic profiling (CGAT, conventional karyotyping, and FISH) in the workup of ALL, providing additional information that aids the risk stratification and understanding of ALL pathobiology. The anchored multiplex technology allowed for the detection of novel fusions, hotspot mutations, and expression changes which indicated potential Ph‐like ALL. RNA sequencing with FusionPlex has a library preparation time of 28 hours (or in a clinical lab with regular work hours of three technologists shifts), a software analysis time of <8 h per run, and reporting potentially performed in 1 day. Therefore, a total projected turnaround time of 5–7 working days to obtain fusion gene results and some common mutations validated on the platform, whereas karyotype analysis typically takes 5–10 working days (although stat karyotype analysis may be completed within 48 h). However, further advantages afforded by implementing RNA sequencing with FusionPlex chemistry include the ability to identify novel fusions without the need for a priori knowledge of fusion genes and their partners.

The feasibility of using fixed cytogenetic cell pellets in an anchored multiplex PCR NGS assay is novel. Our success in utilizing methanol/acetic acid preserved specimens for RNA‐NGS fusion detection enables the use of archived cytogenetic cell pellets when fresh/frozen tissue is not available. Even though an acidic environment has been described previously to cause RNA degradation,^8^ the methanol fixation, along with the storage in −20°C, may have some protective effects on RNA. Overexpression of fusion transcripts along with short‐read lengths required by the NGS method likely compensated for partially degraded RNA. RNA‐based NGS assay is highly sensitive in detecting a given fusion if it leads to the overexpression of the fusion transcript, as exemplified by the detection of *IKZF1* oncogenic isoform in Patient 15, which had merely 2.6% abnormal blasts by flow cytometry at clinical evaluation. Nevertheless, the difference between the gene expression profile of the cryo‐preserved and the paired methanol/acetic acid‐based fixed pellets is seen. We recommend that cytogenetically fixed pellets shall not be used for expression analysis.

Ribonucleic acid (RNA) fusion NGS is complementary with current methods of genetic profiling in the workup of ALL. A significant subset of samples was discordant (8/28 patients) between RNA‐NGS and our current clinical testing techniques such as karyotype, FISH, and CGAT. In studying the discordancy, we revealed several advantages of RNA‐NGS: (a) The anchored multiplex PCR enables the detection of fusions involving promiscuous genes that tend to have multiple known or novel partners as exemplified via *KMT2A* (*MLL*) rearrangements; (b) RNA‐NGS allows for the detection of cryptic rearrangements or fusion genes which are in close proximity to each other thus rendering the inability to visualize by karyotype, such as *STIL*‐*TAL1* and *P2RY8*‐*CRLF2*; (c) *IKZF1* oncogenic isoforms were discordantly detected by RNA‐NGS versus CGAT. RNA‐NGS and CGAT are complementary in detecting *IKZF1* deletion, such that RNA‐NGS is more sensitive in detecting deletions that lead to oncogenic isoforms, namely *IKZF1* Δ4‐7, while CGAT had missed several cases in our study with relatively low tumor content. Conversely, CGAT can detect deletions that lead to haploinsufficiency, but NGS cannot detect haploinsufficiency.^9^

The utility of expression and variant data from RNA fusion NGS may be useful but requires more extensive validation prior to clinical implementation. To our knowledge, our study is among the first clinical implementations of an RNA‐based NGS assay that uses fixed cytogenetic preparations as its starting material. This is also the first study that was able to reliably detect fusion gene RNA transcripts by NGS on archival cytogenetic material. The RNA expression and variant data could support the identification of Ph‐like B‐ALL's diverse genetic signature. The expression of Ph‐like genes (such as *CRLF2*, *IKZF1*, *RUNX1*) was increased in three out of four fresh cases that were tested, indicating the possibility that gene expression signatures can be confirmed within Ph‐like ALL. However, a much more extensive validation with a larger series of fresh tissues would be required to confirm the utility of expression profiling. Patients 7 and 14 (leftmost and rightmost, respectively in Figure 2) both lack *BCR*‐*ABL1* fusion, carry an oncogenic *IKZF1* deletion (Table 2), and both had the overexpression of *CRLF2* despite the absence of fusions or point mutations in those genes. This is consistent with prior research delineating *IKZF1*’s role as a repressor for *CRLF2* expression.^10^ Unsurprisingly, the tissue culture process for cytogenetic studies possibly compromises the expression of the essential genes for Ph‐like ALL. RNA expression from cryopreserved tissues can, therefore, identify *CRLF2*‐overexpressed patients whose overexpression arises from other mechanisms of gene regulation,^11^, ^12^ which portends a significantly worse prognosis.^3^ Expression data in RNA sequencing can only be used when acquired from cryopreserved tissue samples (Figure 5).

Analysis of variant data from fusion NGS proved to be challenging due to calls being based on RNA. Inherent errors are a known problem with variant calls based on RNA sequencing,^13^ as is RNA editing.^14^, ^15^, ^16^ Therefore, we only used manufacturer verified hotspots that were validated by ArcherDX and that they had determined were reliable. We also applied a more stringent filter than the minimum recommended by the manufacturer (AO value (≥5), UAO value (≥3)) and visualized each variant reported in this study. However, they are not independently validated with another platform. Of the four patients carrying verifiable variants, three patients carry driver mutations or variants known to cause drug resistance (two patients with JAK2 p.R683G and one patient with ABL1 p.F317L).^17^ A more extensive validation experiment compared against standard DNA NGS is required before clinical implementation of variant calls based on RNA sequencing is possible. Although more work is needed to expand the library of reliable RNA sequencing variants that can be detected by this platform, its clinical utility is inherent when the same assay can detect not only fusions but also drug‐resistant variants.

We conclude that anchored multiplex PCR RNA sequencing is a good technology for detecting novel oncogenic fusions and isogenic isoforms. Our study demonstrated that, among karyotype, CGAT and targeted RNA sequencing, the abnormal result of one test does not exclude the necessity of the other tests but rather augment the diagnostic workup as a whole. Therefore, for clinical diagnosis of ALL patients, in addition to standard single‐target tests such as RT‐PCR for *BCR*‐*ABL1*, we recommend concurrent karyotype, CGAT, and targeted RNA sequencing studies to identify key diagnostic and prognostic markers as well as aberrations that guide therapy. Furthermore, this targeted RNA sequencing platform tested in this study is a good technology for detecting novel fusions and isogenic isoforms. While SNV can be detected, another more rigorous validation for specific variant analysis is recommended to detect specific RNA reads and the significance of these variants.

## ETHICAL APPROVAL STATEMENT

The Institutional Review Board at the Fred Hutchinson Cancer Research Center approved this study.

## INFORMED CONSENT STATEMENT

Written/signed consent in the form of the IRB‐approved consent documents 999.209.4 Consent and Authorization for the Use of Medical Information in Research and 999.209.5 Consent and Authorization for the Use of Leftover Specimens for Research was obtained for all patients in this study.

## AUTHOR CONTRIBUTIONS

Inception of idea and funding support—Min Fang, Cecilia Yeung, Jerry Radich; Data collection and analysis—Cecilia Yeung, Xiaoyu Qu, Olga Sala‐Torra, David Woolston, Min Fang; Statistical analysis—Xiaoyu Qu; Drafting the manuscript—Cecilia Yeung, Xiaoyu Qu, Olga Sala‐Torra, Min Fang; Editing and revising of the manuscript: Cecilia Yeung, Xiaoyu Qu, Olga Sala‐Torra, David Woolston, Jerry Radich, Min Fang.

## CONFLICT OF INTEREST

The authors disclose no conflict of interest.

## Supporting information

Supplementary Material

Supplementary Material

Supplementary Material

## Data Availability

Research data are not shared.

## References

[1] World Health Organization . World Health Organization Classification of Tumors. WHO Classification of Tumours of Haematopoietic and Lymphoid Tissues. 4th ed. Dwerdlow SH, Campo E, Harris NL Jaffe ES, Pileri SA, Stein H, Thiele J, Vardiman JW, eds. Lyon, France: International Agency for Research on Cancer; 2008.

[2] WHO . WHO Classification of Tumours of Haematopoietic and Lymphoid Tissues. Revised 4th ed. Swerdlow SH, Campo E, Harris NL, Jaffe ES, Pileri SA, Stein H, Thiele J, VARDIMAN JW, eds. Lyon, France: International Agency for Research on Cancer; 2017.

[3] Jain N, Roberts KG, Jabbour E, et al. Ph‐like acute lymphoblastic leukemia: a high‐risk subtype in adults. Blood. 2017;129(5):572‐581. 27919910 10.1182/blood-2016-07-726588 PMC5290985

[4] Roberts KG, Li Y, Payne‐Turner D, et al. Targetable kinase‐activating lesions in Ph‐like acute lymphoblastic leukemia. N Engl J Med. 2014;371(11):1005‐1015. 25207766 10.1056/NEJMoa1403088 PMC4191900

[5] Holmfeldt L, Wei L, Diaz‐Flores E, et al. The genomic landscape of hypodiploid acute lymphoblastic leukemia. Nat Genet. 2013;45(3):242‐252. 23334668 10.1038/ng.2532 PMC3919793

[6] Fang M, Becker PS, Linenberger M, et al. Adult low‐hypodiploid acute B‐lymphoblastic leukemia with IKZF3 deletion and TP53 mutation: comparison with pediatric patients. Am J Clin Pathol. 2015;144(2):263‐270. 26185311 10.1309/AJCPW83OXPYKPEEN

[7] ISCN . An International System for Human Cytogenetic Nomenclature (2013). Shaffer LG, McGowan‐Jordan J, Schmid M, eds. Basel, Swizterland: Karger; 2013:140 pp.

[8] Srinivasan M, Sedmak D, Jewell S. Effect of fixatives and tissue processing on the content and integrity of nucleic acids. Am J Pathol. 2002;161(6):1961‐1971. 12466110 10.1016/S0002-9440(10)64472-0 PMC1850907

[9] Caye A, Beldjord K, Mass‐Malo K, et al. Breakpoint‐specific multiplex polymerase chain reaction allows the detection of IKZF1 intragenic deletions and minimal residual disease monitoring in B‐cell precursor acute lymphoblastic leukemia. Haematologica. 2013;98(4):597‐601. 23065506 10.3324/haematol.2012.073965 PMC3659991

[10] Ge Z, Gu Y, Zhao G, et al. High CRLF2 expression associates with IKZF1 dysfunction in adult acute lymphoblastic leukemia without CRLF2 rearrangement. Oncotarget. 2016;7(31):49722‐49732. 27391346 10.18632/oncotarget.10437 PMC5226542

[11] Dovat S, Ge Z, Song CH, Payne KJ. Regulation of CRLF2 oncogene expression by the Ikaros tumor suppressor in B cell acute lymphoblastic leukemia that occurs at high frequency in Hispanic children. Cancer Epidemiol Biomarkers Prev. 2017;26(2):2.

[12] Song CH, Ge Z, Payne KJ, Dovat S. Epigenetic regulation of CRLF2 oncogene expression by Casein Kinase II (CK2) signaling in B‐cell acute lymphoblastic leukemia that occurs at high frequency in Hispanic children. Cancer Epidemiol Biomarkers Prev. 2018;27(7):179.

[13] Robert C, Watson M. Errors in RNA‐Seq quantification affect genes of relevance to human disease. Genome Biol. 2015;16:177. 26335491 10.1186/s13059-015-0734-x PMC4558956

[14] Furey TS, Diekhans M, Lu Y, et al. Analysis of human mRNAs with the reference genome sequence reveals potential errors, polymorphisms, and RNA editing. Genome Res. 2004;14(10B):2034‐2040. 15489323 10.1101/gr.2467904 PMC528917

[15] Hong JH, Ko YH, Kang K. RNA variant identification discrepancy among splice‐aware alignment algorithms. PLoS One. 2018;13(8):e0201822. 30071094 10.1371/journal.pone.0201822 PMC6072070

[16] Bakhtiarizadeh MR, Salehi A, Rivera RM. Genome‐wide identification and analysis of A‐to‐I RNA editing events in bovine by transcriptome sequencing. PLoS One. 2018;13(2):e0193316. 29470549 10.1371/journal.pone.0193316 PMC5823453

[17] Kearney L, Gonzalez De Castro D, Yeung J, et al. Specific JAK2 mutation (JAK2R683) and multiple gene deletions in Down syndrome acute lymphoblastic leukemia. Blood. 2009;113(3):646‐648. 18927438 10.1182/blood-2008-08-170928

