# Supplementary material for: Mutational profiling in acute lymphoblastic leukemia by RNA sequencing and chromosomal genomic array testing
Source: Cancer Med. 2021 Jul 20;10(16):5629–42. doi: 10.1002/cam4.4101 (PMC8366081; doi:10.1002/cam4.4101)
Supplement: Supplementary file 1 — Supplementary Material [file CAM4-10-5629-s001.docx]

**Supporting Material 1: List of genomic regions targeted in the ALL FusionPlex panel with primers sets designed to detect gene fusions.**

| **ALL fusionplex targets (2018)** |
| --- |
| ABL1 NM_005157 1, 2, 3, 4, 5 Fusion 5' |
| ABL2 NM_005158 2, 3, 4, 5, 6, 7, 8 Fusion 5' |
| BCL11B NM_138576 3, 4 Fusion 5' |
| BCL11B NM_138576 2, 3 Fusion 3' |
| BCL2 NM_000633 3 Fusion 3' |
| BCL2 NM_000633 2 Fusion 5' |
| BCL6 NM_001706 2, 3 Fusion 5' |
| BCR NM_004327 1, 2, 3, 8, 12, 13, 14, 15, 16 Fusion 3' |
| CHD1 NM_001270 1,2 Fusion 5' |
| CREBBP NM_004380 2, 3, 4, 5, 6 Fusion 5' |
| CRLF2 NM_022148 1 Fusion 5' |
| CSF1R NM_005211 9, 10, 11, 12, 13, 14 Fusion 5' |
| EBF1 NM_024007 10, 11, 12, 13, 14, 15 Fusion 3' |
| EPOR NM_000121 7, 8 Fusion 3' |
| ETV6 NM_001987 1, 2, 3, 4, 5, 6 Fusion 3' |
| ETV6 NM_001987 2, 3, 4, 5, 6 Fusion 5' |
| FGFR1 NM_023110 12, 17 Fusion 3' |
| FGFR1 NM_023110 2, 3, 4, 5, 6, 7, 8, 9, 10, 11, 17 Fusion 5' |
| IKZF1 NM_006060 1, 2, 3 Exon Skipping 3' |
| IKZF1 NM_006060 7, 8 Exon Skipping 5' |
| IKZF2 NM_016260 3, 4 Fusion 3' |
| IKZF3 NM_012481 2, 3, 4, 5, 6, 7 Fusion 3' |
| JAK2 NM_004972 6, 7, 8, 9, 10, 11, 12, 13, 15, 16, 17, 18, 19, 20 Fusion 5' |
| KLF2 NM_016270 2, 3 Fusion 5' |
| KMT2A NM_005933 4, 5, 6, 7, 8, 9, 10, 11, 12, 13, 14, 15, 16, 17, 18, 19, 20, 21, 22, 23, 24, 25, 26, 27, 28, 29, 30, 31, 32, 33, 34, 35 Fusion 3' |
| MLLT4 NM_001040000 2 Fusion 5' |
| MYC NM_002467 1, 2 Fusion 5' |
| NF1 NM_000267 14 Fusion 3' |
| NF1 NM_000267 36 Fusion 5' |
| NOTCH1 NM_017617 24, 25, 26, 27, 28, 29 Fusion 5' |
| NOTCH1 NM_017617 24 Fusion 3' |
| NOTCH1 NM_017617 34 Exon Skipping N/A |
| NTRK3 NM_002530 13, 14, 15 Fusion 5' |
| NTRK3 NM_001007156 15 Fusion 5' |
| NUP214 NM_005085 17, 18, 19 Fusion 5' |
| NUP98 NM_016320 8, 9, 10, 11, 12, 13, 14, 15, 16, 17 Fusion 3' |
| NUP98 NM_016320 12, 13 Fusion 5' |
| P2RY8 NM_178129 1 Fusion 3' |
| PAG1 NM_018440 2 Fusion 5' |
| PAX5 NM_016734 1, 4, 5, 6, 7, 8 Fusion 3' |
| PAX5 NM_016734 6, 7, 8 Fusion 5' |
| **PBX1 NM_002585 1, 2, 3, 4, 5, 6, 7, 8, 9 Fusion 5'** |
| PDCD1LG2 NM_025239 1, 2, 3 Fusion 5' |
| PDCD1LG2 NM_025239 5, 6 Fusion 3' |
| PDGFRA NM_006206 9, 10, 11, 12, 13, 14 Fusion 5' |
| PDGFRB NM_002609 8, 9, 10, 11, 12, 13, 14 Fusion 5' |
| PICALM NM_007166 16, 17, 18, 19 Fusion 3' |
| PTK2B NM_173176 2, 3, 4, 5, 6, 7, 8 Fusion 5' |
| RUNX1 NM_001754 2, 3, 4, 5, 6, 7, 8 Fusion 3' |
| RUNX1 NM_001754 5, 6, 7, 8, 9 Fusion 5' |
| SEMA6A NM_020796 1,2 Fusion 3' |
| SETD2 NM_014159 1, 2, 3, 4, 5, 6, 7, 8, 9, 10, 11, 12 Fusion 3' |
| STIL NM_003035 1, 2 Fusion 3' |
| TAL1 NM_003189 2, 4 Fusion 5' |
| TAL1 NM_001290404 2, 3 Fusion 5' |
| TCF3 NM_003200 11, 12, 13, 14, 15, 16, 17, 18 Fusion 3' |
| TYK2 NM_003331 16, 18 Fusion 5' |
| ZCCHC7 NM_032226 1, 2 Fusion 3' |
| ZCCHC7 NM_032226 2, 3, 4 Fusion 5' |
