# Supplementary material for: Mutational profiling in acute lymphoblastic leukemia by RNA sequencing and chromosomal genomic array testing
Source: Cancer Med. 2021 Jul 20;10(16):5629–42. doi: 10.1002/cam4.4101 (PMC8366081; doi:10.1002/cam4.4101)
Supplement: Supplementary file 2 — Supplementary Material [file CAM4-10-5629-s002.pdf]

Supporting Material 2: PreSeq RNA QC Assay Ct values and library yield for the paired frozen and fixed samples

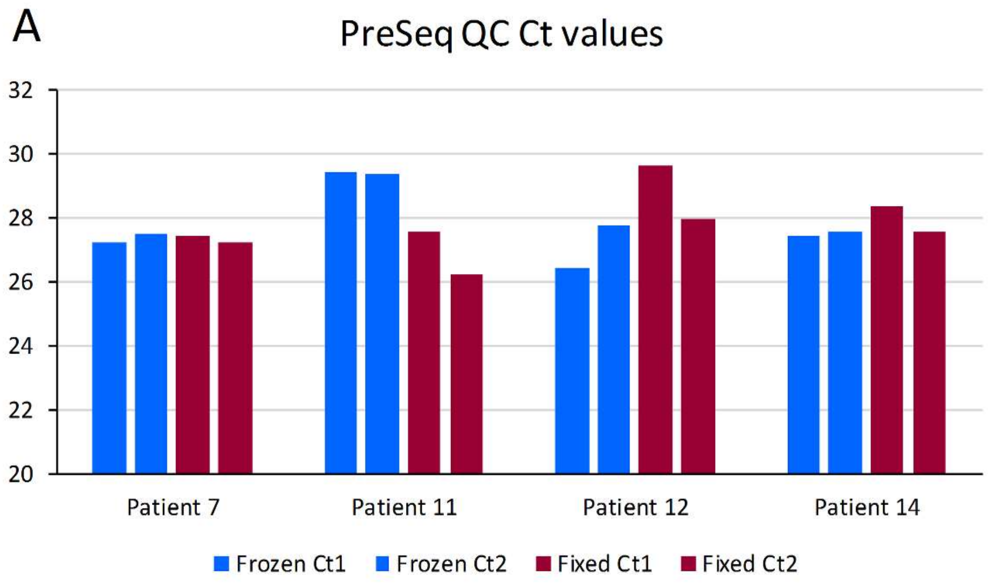

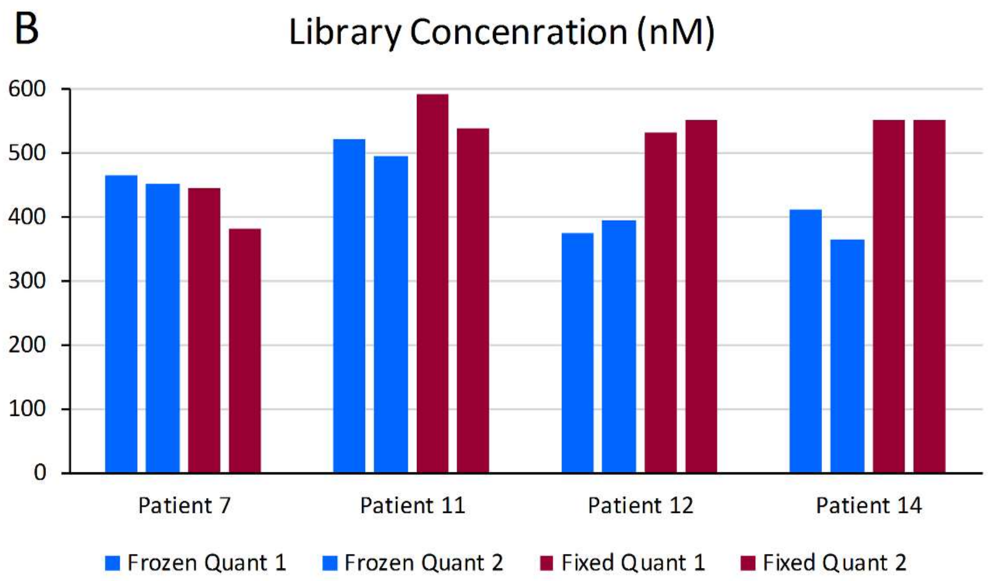

Supporting Material 2 shows that the paired frozen vs. fixed samples had comparable quality metrics: (A) PreSeq Ct values measured using the Archer PreSeq RNA QC Assay, a quantitative polymerase chain reaction (qPCR)-based method to determine the quality of input RNA prior to library preparation; and (B) Library concentration quantified using the KAPA Library Quantification Kits (Roche).
