# Supplementary material for: Mutational profiling in acute lymphoblastic leukemia by RNA sequencing and chromosomal genomic array testing
Source: Cancer Med. 2021 Jul 20;10(16):5629–42. doi: 10.1002/cam4.4101 (PMC8366081; doi:10.1002/cam4.4101)
Supplement: Supplementary file 3 — Supplementary Material [file CAM4-10-5629-s003.docx]

**Supporting Material 3: Verified detectable RNA variants**

| **Gene** | **Accession** | **Description** |
| --- | --- | --- |
| *ABL1* | NM_005157 | Y253-E255, V299, T315-F317, M351-F359 |
| *ALK* | NM_004304 | T1151-C1156, F1174, L1196-S1206, G1269 |
| *BCL2* | NM_000633 | F104 |
| *BIRC3* | NM_001165 | Q547 |
| *CCND1* | NM_053056 | E36, V42-C47 |
| *CEBPA* | NM_004364 | P23-H54, Q83, K304-L317 |
| *CREBBP* | NM_004380 | P1053, C1240, R1446, S1680-L1681 |
| *CRLF2* | NM_022148 | F232 |
| *ETV6* | NM_001987 | Y104-R105 |
| *IKZF3* | NM_012481 | L162 |
| *JAK2* | NM_004972 | F537-F547, V617-C618, L681-R683, L855, V863, A880, V911, M929-R938, I960, R980-E985, D994 |
| *NOTCH1* | NM_017617 | L1574, V1578, L1585, F1592-L1593, R1598-L1600, L1678-I1680, P2514-E2515, P2525 |
| *PAX5* | NM_16734 | P80 |
| *PDGFRA* | NM_006206 | T674 |
| *PML* | NM_002675 | C212-S220 |
| *RARA* | NM_000964 | E197, R272, T283-M284, L290-M297, R394, Q411 |
| *TYK2* | NM_003331 | W327 |

Supporting Material 3 lists the verified RNA variants that can be detected on the FusionPlex Heme v2 RNA sequencing assay per manufacturer’s product insert.
